# 2024 Literature Review of Postoperative Atrial Fibrillation: A Surgeon’s Perspective

**DOI:** 10.19102/icrm.2025.16017

**Published:** 2025-01-15

**Authors:** Andy C. Kiser

**Affiliations:** 1 Cardiovascular Services, St. Clair Health, Pittsburgh, PA, USA; 2 Department of Cardiothoracic Surgery, University of Pittsburgh School of Medicine, Pittsburgh, PA, USA

**Keywords:** Anticoagulation, postoperative atrial fibrillation, prophylactic treatment, risk factor

## Background

Atrial fibrillation (AF) commonly occurs in patients undergoing cardiac surgery, arising in 20%–50% of patients.^1,2^ Postoperative AF (POAF) occurs during the immediate period after cardiac surgery and, although usually transient and self-limiting, may persist long term in up to 25% of patients.^3,4^ POAF is not benign. A meta-analysis of patients with POAF compared to those without POAF demonstrated a 62% higher rate of stroke within 30 days of cardiac surgery and a 37% higher risk of long-term stroke in those patients with POAF.^5^ Over the past year, a series of publications identified risk factors for POAF and discussed therapeutic options such as anticoagulation and prophylactic surgical ablation, which we will examine in this review of the 2024 literature.

## Risk factors of postoperative atrial fibrillation

Bowdish et al.^6^ published a large, multicenter, randomized trial conducted by the Cardiothoracic Surgical Trials Network between 2014 and 2015 examining the risk factors for POAF. This ancillary study included 2104 patients undergoing various cardiac surgeries, including isolated coronary artery bypass grafting (CABG), valve surgery, or combined CABG and valve surgery, while excluding patients with a history of AF. POAF was defined as >60 min of hemodynamically stable AF or at least two episodes of AF within 7 days of surgery. Continuous and categorical variables were presented, and unadjusted odds ratios (ORs) were used to determine univariate predictors associated with POAF. A multivariable logistic regression analysis also explored variables associated with POAF.

POAF occurred in 33% of patients, and the incidence varied by type of procedure, as follows: 28.1% for isolated CABG, 33.7% for isolated valve repair/replacement, and 47.3% for combined CABG and valve repair/replacement. The univariate analysis demonstrated a significant relationship of POAF with older age (68.1 vs. 62.8 years; *P* < .0001), white (93.7% vs. 89.9%; *P* = .002) or non-Hispanic status (96.7% vs. 93.7%; *P* = .004), and preceding heart failure (14.4% vs. 9.4%; *P* = .001). Patients with POAF were also more likely to have hypothyroidism (12.7% vs. 8.6%; *P* = .003). Compared to stand-alone CABG procedures, POAF was seen more frequently in those patients undergoing isolated valve procedures (unadjusted OR, 1.29; *P* = .01) or a combination of CABG and valve procedures (unadjusted OR, 2.29; *P* < .001). In the multivariate logistic regression analysis, each of these variables persisted as statistically significantly associated with POAF.

Established risk factors associated with POAF, such as age, race, heart failure, and hypothyroidism, align with the findings in other studies.^7–10^ Additionally, more complex surgical procedures carry greater risks of POAF.^11^ However, unlike in previous reports,^12,13^ rate- and rhythm-controlling medications and left atrial size were not associated with POAF in this evaluation. The identification of important risk factors may help providers anticipate and implement treatment strategies prophylactically or earlier in the postoperative course.

## Prophylactic treatment of postoperative atrial fibrillation

Willekes et al.^14^ published a single-center feasibility trial of 62 patients without prior dysrhythmias who were randomized to undergo either standard cardiac surgery (control) or surgery with bilateral pulmonary vein isolation (PVI) and left atrial appendage (LAA) amputation (treatment). The study included patients >70 years of age undergoing CABG or aortic valve replacement and excluded patients with a low ejection fraction (<35%) or an enlarged left atrium. PVI utilized a bipolar radiofrequency clamp (AtriCure, Mason, OH, USA) for three separate lesions encircling the left and right pulmonary veins with three sequential applications of energy to each lesion. The LAA was amputated at the base and closed with a 4.0 Prolene suture (Johnson & Johnson, New Brunswick, NJ, USA). Continuous 24-h telemetry until discharge tracked episodes of AF or flutter lasting >30 s.

The incidence of POAF was significantly lower in the treatment (n = 29) group compared to the control (n = 31) group (7% vs. 55%; *P* < .001). Although one patient in the control group had a stroke and one patient in the treatment group required reoperation for a delayed postoperative bleed, no patients experienced a complication directly related to excision of the LAA or required a permanent pacemaker. In the control group, 45% required anti-arrhythmic medications compared to 7% in the treatment group (*P* < .001). At 1 year of follow-up, all patients in the treatment group remained in sinus rhythm and off oral anticoagulation (OAC), while three patients in the control group continued to have AF/flutter and 19% remained on OAC.

The authors highlight the importance of LAA amputation as a contributing factor to successful dysrhythmia prophylaxis. In addition to a reduction in embolic stroke risk,^14,15^ up to 8% of AF triggers may harbor in the LAA.^16,17^ The additional operative and cross-clamp times in the treatment group did not add morbidity or adverse sequelae. Secondarily, the decreased use of anticoagulation in the treatment group may portend reduced risks of bleeding complications.^18–20^ Although ongoing trials may provide more insight, current guidelines recommend anticoagulation, even after LAA exclusion or amputation.^21^

## Anticoagulation for postoperative atrial fibrillation in coronary artery bypass grafting patients

A notable meta-analysis of 28 articles describing POAF after CABG and the use of OAC was performed by van de Kar et al.^22^ After a comprehensive search of MEDLINE, Embase, and the Cochrane Library, 1,698,307 patients comprised the study population. Demographic data as well as POAF incidence, OAC therapy, thromboembolic (TE) and major bleeding rates, and mortality were included in the analysis.

Patients post-CABG had a 23.7% (7.9%–37.6%) mean incidence of POAF, and an average of 15.5% (3.6%–53.0%) received OAC. **Table 1** presents the TE and mortality rates during the index hospital admission, within 30 days, and within 1 year. When extrapolated to examine event rates per 100 patient-years, TE (1.73 vs. 1.14), mortality (3.39 vs. 2.19), and bleeding (2.00 vs. 1.60) rates were higher in patients with POAF than those who did not have POAF. The meta-analysis stratified for OAC use did not demonstrate a significant relationship to TE or mortality rates but did demonstrate a significant difference in bleeding rates favoring no use of OAC for POAF.

**Table 1:** Thromboembolic and Mortality Outcomes in Patients with and Without Postoperative Atrial Fibrillation

	Overall Rates (%)		Thromboembolic Rates (%)		Mortality Rates (%)	
	Thromboembolic	Mortality	POAF	Non-POAF	POAF	Non-POAF
During hospital admission	1.6	1.4	2.6	1.2	2.0	1.2
Within 30 days	1.0	2.0	0.3	0.8	1.0	0.5
Within 1 year	0.6	3.8	0.8	0.5	6.4	2.7

*Abbreviation:* POAF, postoperative atrial fibrillation.

The authors emphasize that TE events more commonly occurred during the index admission, while bleeding events were more commonly encountered within 30 days of surgery. The higher bleeding risk overall for patients on OAC, and the limited reduction in TE events and mortality for patients on OAC, suggests that OAC therapy may not be warranted. However, the authors emphasize that this meta-analysis specific to CABG patients shows significant variability in study designs, patient populations, and anticoagulation regimens across the studies examined, which could limit the generalizability of the findings. More robust, prospective trials for OAC therapy in POAF CABG patients may provide sufficient data for evidence-based decisions.

## Author’s comments

POAF commonly occurs after cardiac surgery, yielding implications of increased morbidity and costs. The identification of clinical risk factors (age, race, heart failure) and an appreciation of the impact of the specific surgical procedure (CABG, valve, or more complex intervention) may encourage more aggressive management strategies in higher-risk populations. Indeed, patients with POAF who undergo valve surgery have almost a two-fold higher risk of stroke than isolated CABG patients (8.0% vs. 2.7%).^23^ However, POAF usually presents during the index hospital admission and resolves within 24 h, with close to 90% of patients in normal rhythm at discharge.^23^

Risk factors for POAF alone do not carry clear indications for a presumptive therapy. While the treatment of concomitant AF during first-time cardiac surgery has a class 1-B indication,^21^ we have little evidence supporting a concomitant prophylactic ablation strategy for those patients without a history of AF. Willekes et al. concluded that prophylactic PVI and LAA exclusion decreases the risk of POAF, but the limited number of patients in the evaluation and the lack of stringent rhythm follow-up do not provide ample evidence for a preventative ablation strategy in this single-center, non-randomized feasibility study.

Furthermore, although obliteration of the LAA during cardiac surgery for patients with AF has a class 1-A indication,^21^ we find little evidence to support the concomitant prophylactic closure of the LAA in patients without a history of AF. In fact, Ascaso et al.^24^ retrospectively examined 1050 patients undergoing mitral valve repair for leaflet prolapse who had concomitant LAA exclusion (n = 269) or no LAA intervention (n = 718). The authors demonstrated no significant difference in stroke or transient ischemia events at 5 years. Even though many surgeons address the LAA during concomitant cardiac procedures for patients in normal rhythm, we have no conclusive evidence that this reduces long-term neurological events.

Anticoagulation of POAF patients also lacks clear and convincing evidence. The findings by van de Kar et al. align with those of other retrospective and registry studies, which generally found limited evidence for stroke and mortality rate reductions but increased bleeding complications.^23^ Oral anticoagulants included both vitamin K antagonists (VKAs) and direct oral anticoagulants (DOACs) in the paper by van de Kar et al. Koh et al.^25^ reported a review of 12 studies comparing DOACs to VKAs (5 randomized and 7 observational) that included 8587 patients with AF. Overall, 7.3% of patients experienced bleeding events and 2.2% suffered major neurological events. In the comparative meta-analysis of DOACs and VKAs, the relative risk (RR) for bleeding complications (RR, 0.74; *P* = .01) and major neurological events (RR, 0.63; *P* = .01) favored DOACs over VKAs, with no significant difference in mortality between the groups. Although DOACs seemed to demonstrate advantages in stroke reduction and bleeding complications, conclusive evidence remains unclear. The ongoing trial Anticoagulation for New-Onset Post-Operative Atrial Fibrillation after CABG (PACES) randomizes patients to either antiplatelet therapy alone or antiplatelet therapy with either DOACs or VKAs. The results of this prospective study of 3200 accrued patients should provide insight into the optimal OAC therapy and the duration of therapy for patients with POAF.
